# Supplementary material for: Identification of photoperiod-induced specific miRNAs in the adrenal glands of Sunite sheep (Ovis aries)
Source: Front Vet Sci. 2022 Jul 22;9:888207. doi: 10.3389/fvets.2022.888207 (PMC9354845; doi:10.3389/fvets.2022.888207)
Supplement: Supplementary file 1 [file Data_Sheet_1.ZIP › Supplementary file/miRDeep2 file.zip!/miRDeep2 file/Know_Structure/LP42A3/oar-mir-3956.pdf]

miRBase precursor : oar-mir-3956
Total read count : 42
oar-miR-3956-5p read count : 39
oar-miR-3956-3p read count : 3
remaining reads : 0

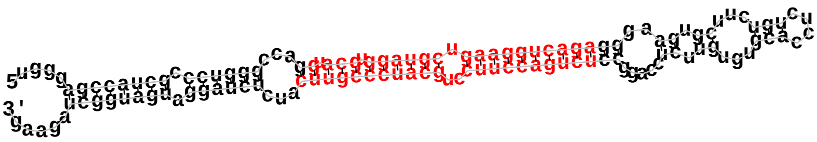

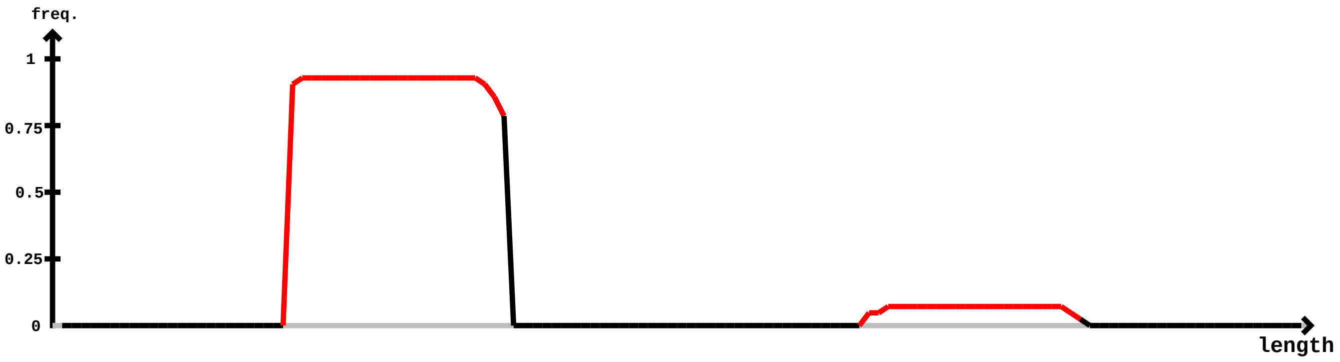


```
                                                                                   oar-miR-3956-3p
                       oar-miR-3956-5p
5'- ugggagccaucgcccugggccagguacguggaugcugaaggucagagggaagugcuucugucuccacguguguucuccaggccucugaccuuccugcaucccguucaucucuaggaugauggcuagaag   -3'   exp
    ....((((((((.((((((...((.(((.((((((.((((((((((((..((.((...(((....)))...)).)).....))))))))))))..)))))))))))...)))))).))))))))).....     reads   mm
    .......................guacguggaugcugaagguc......................................................................................     1       0
    .......................guacguggaugcugaagguca.....................................................................................     2       0
    .......................guacguggaugcugaaggucag....................................................................................     3       0
    .......................guacguggaugcugaaggucaga...................................................................................     32      0
    ........................uacguggaugcugaaggucaga...................................................................................     1       0
    ...................................................................................ucugaccuuccugcaucccgu.........................     1       0
    ...................................................................................ucugaccuuccugcaucccguu........................     1       0
    .....................................................................................ugaccuuccugcaucccguuc.......................     1       0
```
